# Profiles of Killer Systems and Volatile Organic Compounds of Rowanberry and Rosehip-Inhabiting Yeasts Substantiate Implications for Biocontrol

**DOI:** 10.3390/foods14020288

**Published:** 2025-01-16

**Authors:** Iglė Vepštaitė-Monstavičė, Juliana Lukša-Žebelovič, Violeta Apšegaitė, Raimondas Mozūraitis, Robertas Lisicinas, Ramunė Stanevičienė, Laima Blažytė-Čereškienė, Saulius Serva, Elena Servienė

**Affiliations:** 1 Life Sciences Center, Vilnius University, Saulėtekio av. 7, 10257 Vilnius, Lithuania; igle.vepstaite-monstavice@gamtc.lt (I.V.-M.); saulius.serva@gf.vu.lt (S.S.); 2 Nature Research Centre, Akademijos str. 2, 08412 Vilnius, Lithuania; juliana.luksa@gamtc.lt (J.L.-Ž.); violeta.apsegaite@gamtc.lt (V.A.); raimondas.mozuraitis@gamtc.lt (R.M.); rlisicinas@gmail.com (R.L.); ramune.staneviciene@gamtc.lt (R.S.); laima.blazyte@gamtc.lt (L.B.-Č.); 3 Department of Zoology, Stockholm University, Svante Arrheniusväg 18B, 10691 Stockholm, Sweden

**Keywords:** biocontrol, killer yeasts, double-stranded RNA, volatile organic compounds

## Abstract

Yeasts produce numerous antimicrobial agents such as killer toxins, volatile organic compounds (VOCs), and other secondary metabolites, establishing themselves in developing natural and sustainable biocontrol strategies for agriculture and food preservation. This study addressed the biocontrol potential of yeasts, isolated from spontaneous fermentations of rosehips (*Rosa canina* L.) and rowanberries (*Sorbus aucuparia* L.), focusing on their killer phenotypes and VOCs production. Yeasts were isolated using spontaneous fermentations with *Hanseniaspora uvarum* and *Metschnikowia pulcherrima* identified as the dominant species, comprising approximately 70% of the yeast population. Among 163 isolated strains, 20% demonstrated killing activity, with *Saccharomyces cerevisiae* exhibiting the strongest killing efficiency, as well as *Pichia anomala* and *M. pulcherrima* showing broad-spectrum antagonistic activity. This study identified dsRNA-encoded killer phenotypes in *S. cerevisiae*, *S. paradoxus*, and *Torulaspora delbrueckii*, revealing multiple distinct killer toxin types. The biocontrol potential of wild berry-inhabiting yeasts was demonstrated in a real food system, grape juice, where the *S. cerevisiae* K2-type killer strain significantly reduced fungal contaminants. The selected *H. uvarum*, *M. pulcherrima*, *S. cerevisiae*, and *S. paradoxus* yeast strains representing both berries were applied for VOC analysis and identification by gas chromatography-linked mass spectrometry. It was revealed that the patterns of emitted volatiles are yeast species-specific. Statistically significant differences between the individual VOCs were observed among killing phenotype-possessing vs. non-killer *S. paradoxus* yeasts, thus revealing the involvement of killer systems in multi-level biocontrol enablement. The performed studies deepen our understanding of potential yeast biocontrol mechanisms, highlight the importance of produced antimicrobials and volatiles in ensuring antagonistic efficacy, and prove the relevance of isolated biocontrol yeasts for improving food safety.

## 1. Introduction

Yeasts are ubiquitous microorganisms playing crucial ecological roles across various natural environments, including plants and fruits. They are integral to nutrient cycling, engage in symbiotic relationships, and can act as antagonists to other microbes [1,2]. Recently, there has been increasing interest in the use of naturally occurring yeasts for biocontrol applications in agriculture and food preservation, as yeasts offer safer, eco-friendly alternatives to synthetic chemicals [1,3,4]. Residing yeasts act as biological control agents by emitting antimicrobial compounds, such as proteinaceous toxins, volatile organic compounds (VOCs), secondary metabolites, and others [4,5,6]. Accordingly, yeasts associated with wild berries represent an underexplored resource for natural biocontrol, with implications for food preservation and human health.

Wild berries often thrive in harsh environments, being exposed to various stresses such as fluctuating temperatures, limited nutrients, and microbial competition. The challenging conditions may promote the evolution of yeast strains with unique traits that confer survival advantages in such environments [7]. Different yeast species with biocontrol features have been isolated from agricultural fruits and berries [4,5]; however, studies on the appearance of wild berry-associated biocontrol ability-possessing microorganisms are limited. Wild berries, such as rowanberries (*Sorbus aucuparia* L.) and rosehips (*Rosa canina* L.), harbor diverse epiphytic and endophytic fungal communities. Epiphytic yeasts such as *Aureobasidium*, *Cryptococcus*, *Leucosporidium*, *Metschnikowia*, *Rhodotorula*, *Sporobolomyces*, *Sporidiobolus*, *Vishniacozyma*, *Filobasidium*, *Hanseniaspora*, *Curvibasidium*, and *Dioszegia* or endophytic fungal microorganisms such as *Alternaria*, *Aspergillus*, *Candida*, *Cladosporium*, *Epicoccum*, *Phoma*, *Penicillium*, *Rhodotorula*, *Mucor*, *Phoma*, and *Trichoderma* were observed on both berries [8,9,10]. These yeasts hold the potential for reducing microbial spoilage and pathogenic interactions in crops, aiding in the sustainable production and preservation of food products.

One of the most potent biocontrol mechanisms observed in yeasts is determined by killer phenotype—an ability to produce toxins that inhibit or kill other microorganisms. Killer phenotype can be encoded in a chromosome, linear DNA plasmid, or double-stranded RNAs replicated and encapsulated by *Totiviridae* family viruses [11]. L-A dsRNA encodes for RNA-dependent RNA polymerase and coats proteins, while satellite M dsRNA comprises information on toxins and immunity. Toxins originating from dsRNAs are produced by a variety of yeast species, including *S. cerevisiae*, *S. paradoxus*, *S. uvarum*, *S. mikatae*, *S. bayanus*, *S. kudriavzevii*, *H. uvarum*, *T. delbrueckii*, *U. maydis*, *Z. bailii*, *P. membranifaciens*, and *M. pulcherrima* [11,12,13,14,15]. Most dsRNA-encoded killer toxins have been identified in *S. cerevisiae* (K1, K2, K28, and Klus) and *S. paradoxus* (K1L, K21, K45, K62, K66, and K74) yeasts [11,13,14,15,16,17,18]. Each type of killer toxin differs by the structure and mode of the action. Some toxins exert their lethal effect by disrupting target cell membrane functions and causing uncontrolled ion leakage [19,20,21,22]. In contrast, others transduce toxic signals into the nucleus by inhibiting DNA synthesis and arresting yeast budding [23,24]. Killer toxin-driven biocontrol is directed against other yeasts and gives the host an advantage in competition for nutrients and space, and it is attractive in the food and fermentation industries to prevent spoilage [11,25,26,27,28].

In addition to the killer phenotype, yeasts produce a range of volatile organic compounds (VOCs), which exhibit antimicrobial activities across a broad spectrum of pathogens [5,29,30,31,32]. VOCs, released as part of yeast metabolism, vary in chemical composition depending on the yeast species, environmental factors, and pathogens being antagonized [2,33,34,35,36]. So far, VOCs produced by *Pichia kudriavzevii*, *P. kluyveri*, *P. anomala*, *P. fermentans*, *P. membranifaciens*, *H. uvarum*, *Cr. wieringae*, *S. paradoxus*, *T. delbrueckii*, *M. pulcherrima*, *A. pullulans*, *Candida intermedia*, and *Starmerella bacillaris* yeasts isolated from sour and sweet cherries, sea buckthorns, strawberries, and grapes have been studied [34,35,37,38,39]. The biocontrol of yeasts producing VOCs is an effective antifungal strategy against *Botrytis cinerea*, *Colletotrichum acutatum*, *Penicillium expansum*, *Penicillium digitatum*, *P. italicum*, *Monilinia* spp., *Alternaria* spp., *Aspergillus* spp., *Fusarium* spp., *Geotrichum* spp., *Gloeosporium* spp., *Rhizopus* spp., etc. [5,37,39,40]. VOCs can be regarded as effective antimicrobials since this activity does not require direct contact between the biocontrol agent and the pathogen or the food product [33]. Volatile compounds are suitable for biofumigation, where they are used to control pests and diseases from beneficial microorganisms in confined environments such as storage facilities or shipping containers [32].

Yeasts can employ several biocontrol mechanisms simultaneously to enhance bioactivity; however, the information on the interactions of different biocontrol modes is limited [2]. The relationship between yeast killer toxins and VOCs can be inferred due to sharing similar functions, such as an antagonistic effect against other microorganisms. Both the execution of the killer phenotype and the production of VOCs depend on hosting yeast species and their metabolic activity, surrounding environment, and target microorganisms [4,41,42,43]. The functioning of yeast killer systems impacts the host gene expression pattern, affects cellular processes, and may alter VOC profiles [44]. The presence of killer strains in co-cultures can change metabolite production and have an impact on bioprotection [41]. On the other hand, VOCs can inhibit or activate toxin production, thus changing the microbial population in the surrounding environment [42]. It should be noted that yeast-secreted killer toxins execute their action in a limited space through direct contact with the target, while VOCs can diffuse to a greater distance in a structurally heterogeneous environment and affect the survival of the other microorganisms. Therefore, both modes may complement each other and enhance the antagonistic effect [2,43].

Wild berries provide a promising and largely untapped resource for biocontrol yeasts, naturally coexisting with plants and protecting them from harmful microorganisms. Therefore, for screening and analyzing attractive biocontrol feature-possessing yeasts, we choose rosehips and rowanberries, which grow in natural and challenging chemical-free environments. It must be noted that the metagenomic analysis of microbial communities distributed on the surface of tested wild berries is available [8]; however, the peculiarities of the mechanisms potentially involved in the bioactivity of these berries inhabiting yeasts are yet to be uncovered. Therefore, the aim of this study was to comprehensively analyze rosehips and rowanberry-associated yeasts by revealing the importance of the killing activity and production of VOCs for biocontrol, as well as deepen insight into the potential relationship between different biocontrol modes. In this context, the objectives were (i) to isolate and identify cultivable yeasts from the spontaneous fermentations of rosehips and rowanberries; (ii) to evaluate their antagonistic activity and reveal dsRNA-encoded killer phenotype-possessing yeasts; (iii) to investigate the VOC profiles emitted by different killer toxin-producing and susceptible yeast species; and (iv) to demonstrate the biocontrol potential of wild berry-inhabiting yeasts in a real food system. The targeted investigation of promising biocontrol yeasts inhabiting rosehips and rowanberries was performed for the first time by linking VOC profiles emitted by different yeast species and the occurrence of the killer phenotype. The performed studies deepen insight into potential yeast biocontrol mechanisms and their relevance for ensuring plant and human health. The isolated yeast strains with described unique species-related volatile profiles and efficient killing properties could be employed as natural biocontrol tools in the food industry and agriculture.

## 2. Materials and Methods

### 2.1. Yeast Cultures and Media

Yeast strains analyzed in this study (*M. pulcherrima* SA-5-25.1, SA-5-25.3, SA-7-70, RC-2-2, RC-6-27, RC-6-67; *H. uvarum* SA-4-20, SA-4-33, SA-4-52, RC-4-4, RC-6-36, RC-4-46; *S. paradoxus* SA-4-10, SA-7-12, SA-5-26, RC-2-17, RC-8-28, RC-2-40; *S. cerevisiae* SA-5-8, SA-6-60, SA-N4, RC-3-11, RC-7-34, RC-8-64; *P. anomala* SA-4-39, RC-8-20, RC-2-63; *P. kluyveri* SA-7-30, SA-4-48, RC-6-31; *T. delbrueckii* RC-2-70, RC-2-72, RC-7-72) and were isolated from the spontaneous fermentation of rosehips (RC—*Rosa canina* L.) and rowanberries (SA—*Sorbus aucuparia* L.). Other yeasts were used for the killer assay and biocontrol experiment in grape juice: *S. cerevisiae* strain α’1 (MATα leu2-2 (Kil-0)), K7 (MATα arg9 (KIL-K1)), M437 (*wt*, HM/HM (KIL-K2)), MS300 (MATα leu2 ura 3-52 (KIL-K28)) [45] SRB-15-4 (*wt*, HM/HM (KIL-Klus)) (Nature Research Centre, collection of Laboratory of Genetics), *S. paradoxus* AML-15-66 [16], *M. pulcherrima* 2-34 (K+), 5-47 (K−), *H. uvarum* 3-8 (K−), 1-14 (K−), *P. fermentans* 4-36 (K+), 4-39 (K+), *P. kluyveri* 7-12 (K+), 6-57 (K+), *P. membranifaciens* 8-41 (K+), 8-47 (K+), *T. delbrueckii* 5-17 (K−), 1-61 (K−), *Candida albicans* 21.1, and *P. anomala* 7-13.5 (Nature Research Centre, collection of Laboratory of Genetics).

Yeast strains were grown in YEPD medium: 1% yeast extract, 2% peptone, 2% dextrose, and 2% agar. For identification of the killing phenotype, MBA agar plates (0.5% yeast extract, 0.5% peptone, 2% dextrose, 1.05% citric acid, 3.53% Na_2_HPO_4_ × 12 H_2_O, 2% agar, 0.002% methylene blue dye; adjusted to pH 4.8 with 75 mM phosphate–citrate buffer) were used. For toxin crude extract preparation, liquid SC medium (2% dextrose, 1.29% citric acid, 2.76% Na_2_HPO_4_ × 12 H_2_O, 0.2% K_2_HPO_4_, 0,1% MgSO_4_ × 7 H_2_O, 0.1% (NH_4_)_2_SO_4_, 5% glycerol, pH 4.8) was used. All reagents used for media preparation were of a microbiological grade (Liofilchem S.r.l., Roseto degli Abruzzi, Italy). 

### 2.2. Sampling, Enrichment, and Identification of Cultivable Yeast

Rosehips were harvested in late August 2022 year, and rowanberries were collected in mid-September in the Vilnius district, Lithuania [8]. Visually healthy fruits (30 g) were aseptically collected into sterile Falcon tubes containing 5% dextrose solution for 2 weeks at a temperature of 22 °C. Control samples (5% dextrose solution without fruits) were incubated under the above conditions and analyzed to assess potential contamination. After serial dilutions in Ringer solution (Merck, Kenilworth, NJ, USA), samples were plated on YEPD-agar plates supplemented with 50 µg/mL chloramphenicol and incubated for 2 days at 25 °C. Colonies with distinct morphologies were identified using molecular biology methods. DNA was isolated from fresh yeast cultures using a Genomic DNA purification kit (Thermo Fisher Scientific Baltics, Vilnius, Lithuania). For yeast identification, the region between 18S rRNA and 28S rRNA genes was amplified by PCR using ITS1 (5′-TCCGTAGGTGAACCTGCGG-3′) and ITS4 (5′-TCCTCCGCTTATTGATATGC-3′) primers, or the D1/D2 region of 26S rDNA was amplified using NL1 (5′-GCATATCAATAAGCGGAGGAAAAG-3′) and NL4 (5′-GGTCCGTGTTTCAAGACGG-3′) primers (BaseClear, Leiden, The Netherlands), following a previous study [46,47]. PCR products were purified using the GeneJet PCR purification kit (Thermo Fisher Scientific Baltics, Vilnius, Lithuania) and sequenced at BaseClear (Leiden, The Netherlands). Sequencing results were compared with those found in the FASTA network service of the EMBL-EBI database (https://www.ebi.ac.uk/jdispatcher/sss/fasta/nucleotide, accessed on 2 October 2024) and deposited in the National Center for Biotechnology Information (NCBI) under accession numbers PQ849343-PQ849348, PQ851534-PQ851548.

### 2.3. Analysis of Killing/Susceptibility Phenotype

To detect the killing phenotype, 3 µL of overnight cultures of test strains were spotted onto pH 4.8 MBA agar plates that were pre-seeded with a lawn (2 × 10^6^ cells/plate) of the sensitive *S. cerevisiae* strain α’1. After incubating plates at 25 °C for 2 days, clear zones of growth inhibition around the killer cells were assessed on digitalized images (using the ImageJ software v1.53k) and identified as killer activity [48]. Sensitivity and resistance tests involved spotting tested strains onto MBA plates overlaid with different species of yeast strains, such as *M. pulcherrima*, *H. uvarum*, *S. paradoxus*, *S. cerevisiae*, *P. fermentans*, *P. kluyveri*, *P. membranifaciens*, and *T. delbrueckii*. A resistant phenotype was indicated by the absence of a lysis zone around spotted yeasts, whereas clear zones without yeast growth around colonies producing various killer toxins indicated a sensitive phenotype. All experiments were carried out in triplicate and results presented as mean values of zone of inhibition measurements ± standard deviation.

### 2.4. Double-Stranded RNA Isolation from Yeast

Extraction of RNA from yeast was performed based on previously described methods [16,49,50] with some modifications. The yeast cultures were grown in YEPD medium overnight at 30 °C with shaking at 200 rpm. A 3 mL aliquot of the culture was collected by centrifugation for 2 min at 6000× *g* at room temperature and washed with 1/10 part of the volume of the starting culture of 50 mM EDTA solution. The pellet was resuspended in 1/10 of the starting volume of 50 mM Tris-HCl buffer, pH 9.3, containing 1% (*v*/*v*) 2-mercaptoethanol, and incubated for 15 min at room temperature. The cells were collected by centrifugation at 6000× *g* for 2 min and mixed with 2/10 part of the starting volume of lysis buffer (10 mM Tris-HCl pH 8.0; 100 mM NaCl; 10 mM EDTA; 0.2% SDS) and an equal volume of phenol (pH 4.5–5.0). All reagents were of a molecular biology grade (Carl Roth GmbH & Co. KG, Karlsruhe, Germany). This mixture was incubated for 30 min at room temperature with constant shaking. After incubation, the mixture was centrifuged at room temperature at 20,000× *g* for 5 min. Then, the upper aqueous phase was transferred to a new tube, adding 1/10 volume of 3 M sodium acetate (pH 5.2) and 1 volume of isopropanol. After centrifugation for 5 min at 20,000× *g*, the pellet was washed with 75% ethanol and dissolved in DEPC-treated water. The isolated total nucleic acid fraction was mixed with 1 volume of 5 M LiCl and incubated at 4 °C for 16 h and then centrifuged for 30 min at 20,000× *g* at 4 °C. The supernatant was combined with 1/10 volume of 3 M sodium acetate (pH 5.2) and 1 volume of 96% ethanol and incubated at −20 °C for 30 min. Finally, the sample was centrifuged for 10 min at 20,000× *g*, washed with 75% ethanol, air-dried briefly, and dissolved in DEPC-treated water. To analyze the L and M dsRNA, samples were loaded onto a 1% (*w*/*v*) agarose gel prepared in 1x TAE buffer (40 mM tris, 20 mM acetic acid, 1 mM EDTA, pH 8.0). Electrophoresis was performed at 120 V for 45 min. Nucleic acids were visualized under UV light after staining gel with ethidium bromide. The size of extracted dsRNAs was determined using the GeneRuler DNA ladder mix (Thermo Fisher Scientific, Vilnius, Lithuania), and control dsRNAs were derived from a known type of killer yeast strain. 

### 2.5. Analysis of Biocontrol Properties of Killer Yeasts in Food System

For toxin preparation, the *S. cerevisiae* RC-3-11 strain was grown in SC medium for 4 days at 18 °C until reaching a cell density of approximately 0.6 OD_600_. The cell-free toxin extract was prepared using centrifugation at 3000× *g* for 10 min following filtration of supernatant through 0.22 μm sterile PVDF membrane (Millipore, Bedford, MA, USA).

The green grapes, purchased from a local food store (Vilnius, Lithuania), were washed with tap water and drained. The fresh juice was squeezed, filtered through cotton cloth, and adjusted to pH 4.8. Prepared juice samples were sterilized for 10 min at 80 °C and used for analysis of the biocontrol activity of killer yeast. Overnight-grown tested yeast cells were collected by centrifugation at 6000× *g* for 5 min, washed two times with 0.9% NaCl solution, and resuspended in 0.9% NaCl at a final concentration of about 1 OD_600_. To analyze antifungal activity, yeast cells (50 µL) were mixed with 200 µL of grape juice or SC media, and 200 µL of killer toxin-producing culture filtrate was added. In control samples, toxin preparation was substituted by SC media or grape juice. Samples were incubated at 22 °C temperature for 24 h and 50 µL of each serially diluted solution was spread onto YPD agar plates incubated for 2 days at 30 °C temperature. The surviving yeast colony-forming units (CFUs) were counted and presented as a percentage of the whole yeast population. The average number of viable yeast cells in the control was treated as 100%. All experiments were performed in triplicate, and the data were reported as mean values ± standard deviation. Statistical analysis was performed using one-way ANOVA in Microsoft Excel to assess the significance of differences between treatment groups. Differences were considered statistically significant at *p* < 0.05.

### 2.6. Sampling and Analysis of Volatile Organic Compounds Produced by Yeasts

For a sampling of volatile organic compounds, various yeast strains were selected based on their distinct functional properties: *S. cerevisiae* and *S. paradoxus* strains were selected based on killing phenotype (killer and non-killer strains, respectively), while *M. pulcherrima* and *H. uvarum* were used as yeasts potentially participating in biocontrol [35,36]. Overnight-grown yeast cells (50 µL) at a concentration of approximately 3–5 × 10^7^ cells/mL were spread on the surface of the YEPD medium and incubated for two days at 25 °C. Headspace VOCs produced by the yeast were collected using solid-phase micro-extraction (SPME). Control samples consisted of YEPD plates without yeast to sample background VOCs. The SPME needle was inserted above the yeast culture through a small hole in a Petri dish, and the fiber, coated with polydimethylsiloxane–divinylbenzene (PDMS–DVB, 65 µm coating layer thickness, Supelco, Bellefonte, PA, USA), was exposed to the headspace for 1 h at room temperature. The collected VOCs were desorbed for 2 min in the injection liner of a gas chromatograph (Clarus 500, PerkinElmer, Waltham, MA, USA).

The collected VOCs were analyzed using gas chromatography–mass spectrometry (GC–MS). A DB-Wax column was used to separate the compounds under the following temperature program: initially held at 40 °C for 1 min, then gradually increased to 200 °C at a rate of 5 °C/min, followed by a rise to 240 °C at 10 °C/min, where it was held isothermal for 11 min. The GC injector operated isothermally at 240 °C, with helium as the carrier gas. The relative quantities of each compound were determined by the area of their chromatographic peaks. The identification of the VOCs was based on comparisons of their mass spectra and retention indexes with those in the NIST version 2.0 mass spectral library and synthetic standards. C8–C28 n-alkanes were used to calculate retention indices.

### 2.7. Statistical Analysis

A nonparametric Mann–Whitney U test was applied to evaluate differences in VOC amounts between yeast and control groups. The test was implemented using the mannwhitneyu function from scipy.stats module in Python v3.12.4 [51,52], specifying a two-tailed test with a significance level (α) of 0.05. The Kruskal–Wallis test was employed to assess VOC profile differences across multiple yeast strains using the kruskal function from the same module, with pairwise post hoc comparisons conducted to identify specific group differences [51,52]. Principal component analysis (PCA) was performed using the online tool SRplot [53] to visualize clustering patterns among VOC profiles. The analysis was conducted using the tool’s default settings, with the data log-transformed before analysis to normalize variance and reduce the impact of outliers. The data points were grouped by yeast strain to assess clustering and separations in the principal component space.

## 3. Results

### 3.1. Cultured Yeast in Spontaneous Fermentations of Rowanberries and Rosehips

**Figure 1:**
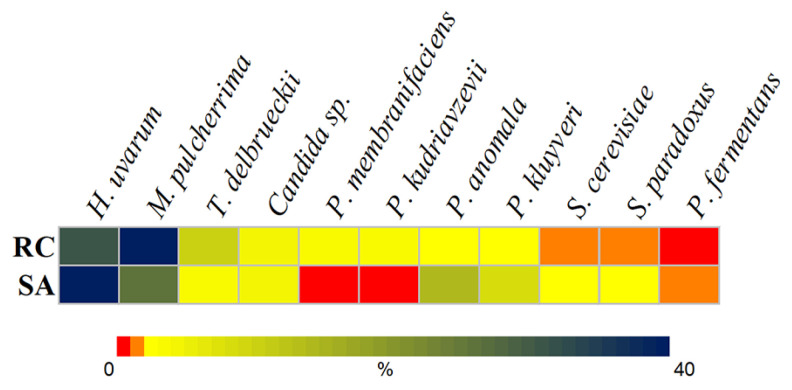
Abundance of cultivable yeast in spontaneous fermentations of rosehips (RC) and rowanberries (SA).

Twenty-eight rowanberry and thirty rosehip samples were collected during late summer and early autumn of 2022. Each sample, consisting of visually healthy and mature berries, was harvested during the peak season to ensure relevant yeast diversity. Berries were fermented in 5% dextrose solution at room temperature for two weeks to promote a diverse range of yeast growth, typically found on berry surfaces, while minimizing bacterial contamination. From the rowanberry samples, 74 yeast isolates representing nine yeast species were obtained, while 89 yeast isolates were cultivated from rosehip samples, representing ten species (each with an abundance greater than 1%) (Figure 1).

The most abundant yeast species in both types of berries were *H. uvarum* and *M. pulcherrima*, known for their dominance in wild fermentations and potential roles in flavor development. Following these species, *P. anomala* (14%) and *P. kluyveri* (8%) were prominent in rowanberry samples, while *T. delbrueckii* was notable in rosehip samples with a relative abundance of 10%. Other yeast species, such as *Candida* spp., *S. cerevisiae*, and *S. paradoxus*, were detected in small amounts on both berries. *P. kudriavzevii* and *P. membranifaciens* were found exclusively in rosehip samples, while *P. fermentans* was isolated from rowanberry samples only.

### 3.2. Antagonistic Activity of Yeasts

**Figure 2:**
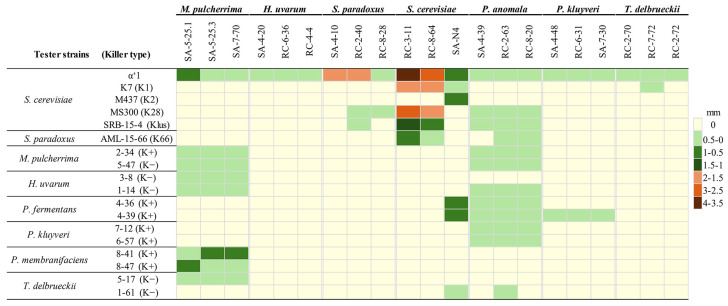
Killing phenotype observed in representative yeast strains. Tester strains were seeded as a lawn in MBA agar plates, and analyzed killer toxin-producing strains were spotted on this monolayer. Plates were grown at 25 °C for 2 days before measurements of growth inhibition zones were taken. Data represent the mean value from three plates.

All yeast cultures were assessed for killer activity against a non-killer *S. cerevisiae* α’1 strain grown on a monolayer at pH 4.8. Thirteen yeast strains from rowanberry and eighteen from rosehips possessed the killing property. Killer yeast strains, representing particular yeast species, are listed in Figure 1. It was determined that *S. cerevisiae* and S. paradoxus yeasts demonstrated the highest killing efficacy (Figure 2, Table S1). *S. cerevisiae* RC-3-11 had the strongest activity (inhibition zone 3.9 ± 0.4 mm) against the non-killer *S. cerevisiae* α’1 strain, followed by *S. cerevisiae* RC-8-64 (2.8 ± 0.3 mm), *S. paradoxus* SA-4-10, and RC-2-40 (lysis zones 1.8 ± 0.2 and 1.9 ± 0.3 mm, respectively). *M. pulcherrima* SA-5-25.1 and *S. cerevisiae* SA-N4 strains were identified as weaker killers (both inhibition zones 0.9 ± 0.1 mm) (Figure 2). The remaining tested strains, representing *H. uvarum*, *M. pulcherrima*, *P. anomala*, *P. kluyveri*, and *T. delbrueckii* yeast species, possessed the lowest killing activity, as evidenced by lysis zones not exceeding 0.5 mm.

Killer yeasts obtained from the spontaneous fermentation of rosehips and rowanberries were evaluated for their antagonistic activity against various killer and non-killer yeast species (Figure 2).

*S. cerevisiae* strains RC-3-11 and RC-8-64 had the strongest antagonistic activity against different types of *S. cerevisiae* killer yeasts. The highest activity was observed against K28 and K1 killer toxin-secreting *S. cerevisiae* strains (the size of lysis zones varied from 1.6 to 2.7 mm), and slightly lower activity was detected through Klus toxin-producing yeasts (1.4 ± 0.3 and 0.9 ± 0.1 mm, respectively). Non-killing activity was identified against the *S. cerevisiae* M437 strain secreting the K2-type toxin, thus indicating that both RC-3-11 and RC-8-64 strains employ a system similar to the M437 killer system, providing resistance to the action of K2 toxin. *S. cerevisiae* SA-N4 strain was the weakest among other *S. cerevisiae* strains tested (formed only a 0.4 ± 0.2 mm lysis zone against K7 and 0.8 ± 0.2 mm against M437 yeasts). However, this isolate had a broader killing spectrum—it can inactivate *P. fermentans* and *T. delbrueckii* species. *S. paradoxus* RC-2-40 isolate exhibited weak antagonistic activity against *S. cerevisiae* K28 and Klus killer strains, while RC-8-28 was active only against the *S. cerevisiae* K28 toxin-producing strain.

*P. anomala* yeasts demonstrated the broadest spectrum of antagonistic activity against *S. cerevisiae*, *S. paradoxus*, *H. uvarum*, *P. fermentans*, *P. kluyveri*, and *T. delbrueckii*. However, the killing efficacy was weak (formed lysis zones did not exceed 0.5 mm). *M. pulcherrima* tested strains exhibited the highest killing activity against P. membranifaciens yeasts (size of lysis zones about 1 mm) and slightly inhibited *M. pulcherrima*, *H. uvarum*, and *T. delbrueckii* strains (lysis zones about 0.5 mm). *P. kluyveri* strains demonstrated weak killing efficacy against *P. fermentans*, while *T. delbrueckii* RC-7-72 demonstrated weak killing efficacy against the *S. cerevisiae* K7 strain only. *H. uvarum* exhibited no antagonistic effects on any target strain, except for the non-killer *S. cerevisiae* α’1 strain.

### 3.3. DsRNA-Encoded Killer Phenotype-Possessing Yeasts

**Figure 3:**
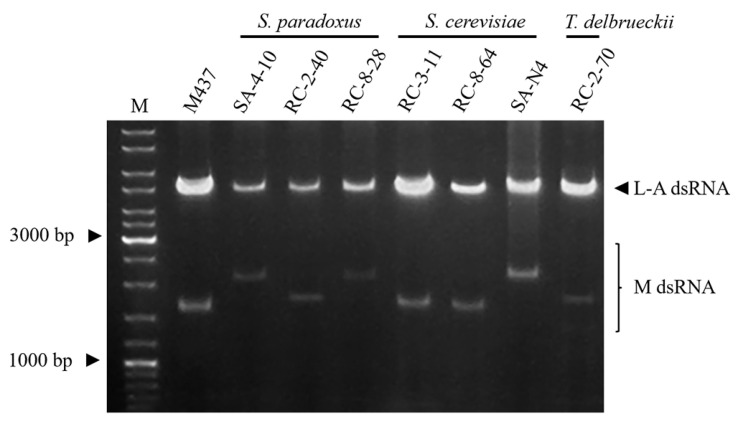
Electrophoretic analysis of L-A and M dsRNAs extracted from killer yeast. M denotes the molecular weight marker GeneRuler DNA Ladder Mix.

To investigate whether the observed killer properties were associated with double-stranded RNA (dsRNA) viruses, nucleic acids were extracted from all killer phenotype-possessing strains and analyzed by agarose gel electrophoresis (Figure 3). Out of the thirty-one yeast strains tested, seven contained dsRNA segments resembling those of *Totiviridae* viruses (about 4.6 kbp), along with smaller satellite dsRNA fractions. In three *S. paradoxus* killer strains, the M dsRNA fractions ranged from 1.8 kbp to 2.2 kbp. The similarity in size (about 1.8 kbp) of M dsRNA from the *S. paradoxus* RC-2-40 strain and K66 toxin-secreting strain AML-15-66 [16], along with the lack of cross-reactivity (Figure 2), suggests that both secrete similar types of killer toxins. While both *S. paradoxus* SA-4-10 and RC-8-28 strains had similarly sized M dsRNAs (about 2.2 kbp) (Figure 3), they varied by antagonistic activity and efficacy (Figure 2), and thus most likely belonged to different killer types.

In *S. cerevisiae* RC-3-11 and RC-8-64 strains, similar to the M437 K2-type, killer strain M dsRNAs (about 1.6 kbp) have been identified. Considering that these strains did not possess antagonistic activity against the K2-type killer system harboring the M437 strain (Figure 2), they likely belong to the same killer type. Based on the electrophoretic analysis, the *S. cerevisiae* SA-N4 strain contained about 2.2 kbp M dsRNA, which was comparable in size to Mlus dsRNA [16]. Among the identified seven dsRNA-encoded killer toxin-secreting strains, only one belonged to *T. delbrueckii* yeast. Based on the size of the killer protein-encoding M dsRNA (approximately 1.7–1.8 kbp), T. delbrueckii strain RC-2-70 may produce similar to T. delbrueckii Kbarr-1 killer toxin [54].

### 3.4. Application of Killer Yeasts in the Food System

**Figure 4:**
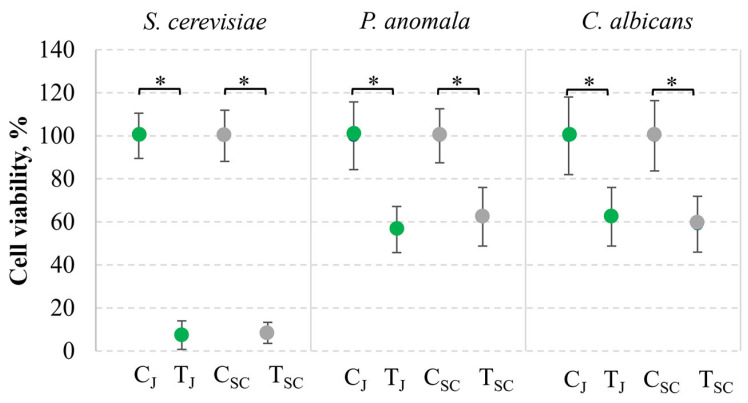
The viability of *S. cerevisiae*, *P. anomala*, and *C. albicans* yeast under the action of yeast killer toxin in grape juice (J) and medium (SC). C—control, yeast cells only; T—yeast cells incubated with killer toxin. Asterisks represent statistically significant differences (*p* ˂ 0.05) between the survival of tested microorganisms in the control and killer toxin-treated samples. Green color indicates juice samples, while gray—SC medium.

The biocontrol potential of the *S. cerevisiae* killer strain RC-3-11, which exhibited the highest antagonistic activity, was analyzed in grape juice (Figure 4). Antimicrobial activity was tested against winemaking and brewing contaminants, including *S. cerevisiae*, *C. albicans*, and *P. anomala.* The most pronounced inhibitory activity was observed against *S. cerevisiae* yeasts, frequently associated with wine refermentation.

The addition of toxin extract into grape juice resulted in a statistically significant reduction of *S. cerevisiae* α’1 cells after 24 h incubation. More than 93% of yeast cells lost viability. In the SC medium, the activity of the killer toxin against *S. cerevisiae* was comparable and led to a decrease in cell viability of up to 91.58%. The lower efficacy of the *S. cerevisiae* RC-3-11 strain-secreted toxin was observed against *C. albicans* and *P. anomala* yeasts. After 24 h treatment, the total viability of tested yeasts was similar in both solutions and accounted for 56–62%. The reduction was statistically significant (*p* < 0.05) compared to the toxin-free samples. The obtained data suggest the promising biocontrol potential of killer yeasts for reducing microbiological contamination in juice.

### 3.5. Volatiles Produced by Different Yeast Species

**Figure 5:**
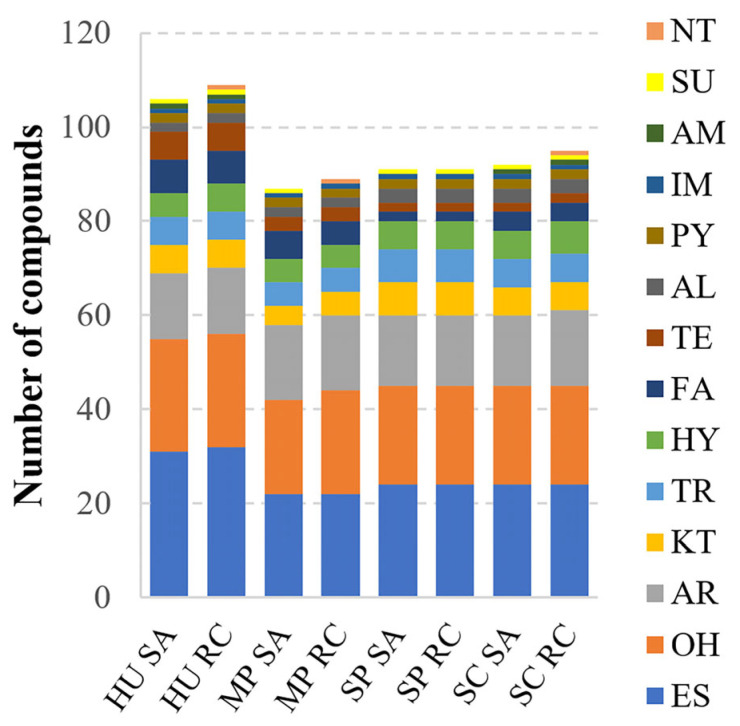
Chemical diversity of volatile organic compounds produced by yeasts. HU—*H. uvarum*, MP—*M. pulcherrima*, SP—*S. paradoxus*, SC—*S. cerevisiae*; SA—rowanberry (*Sorbus aucuparia* L.), RC—rosehip (*Rosa canina* L.). Functional group of volatiles: ES—ester, OH—alcohol, AR—aromatic, KT—ketone, TR—terpen, HY—hydrocarbon, FA—fatty acid, TE—terpenoid, AL—aldehyde, PY—pyrazine, IM—imine, AM—amide, SU—sulfide, NT—nitrile.

Killer and non-killer *S. paradoxus* and *S. cerevisiae* strains, and potential biocontrol yeasts from *H. uvarum* and *M. pulcherrima* species (Figure 2) were chosen for the gas chromatographic–mass spectrometric analysis (GC–MS). One hundred and twenty-six VOCs were collected from the headspace of the four different yeast species isolated from rowanberries and rosehips (Table S2). The most abundant compounds emitted by tested yeasts are presented in Table 1. All identified compounds belonged to 14 groups: esters, alcohols, aromatics, ketones, terpenes, hydrocarbons, fatty acids, terpenoids, aldehydes, pyrazines, imines, amides, sulfides, and nitriles (Figure 5). The composition of volatiles produced by the same yeast species isolated from rowanberries and rosehips was similar, as the profiles differed by one to two VOCs. However, the quantities and profiles of released VOCs varied depending on yeast species. *H. uvarum* produced the most compounds (106–109), followed by *S. cerevisiae* 92–95, *S. paradoxus* 91, and *M. pulcherrima* 87–89.

The most abundant groups of VOCs released by yeasts were esters (ES) and alcohols (OH) (22 to 32 compounds depending on yeast species). The aromatics (AR) were the third most numerous group of VOCs, comprising 14–16 compounds in each yeast species. A similar distribution of ketones (KT), terpenes (TR)**,** and hydrocarbons (HY) (four to seven compounds) was identified in all tested yeast strains. Fatty acids (FA) were less frequently found and differed between yeast species: six FA compounds were detected in *H. uvarum*, five in *M. pulcherrima*, four in *S. cerevisiae*, and two in *S. paradoxus*. Minor VOCs, belonging to aldehyde, pyrazine, imine, amide, sulfide, and nitrile groups, were found only in certain yeast species (Table S2).

Among the individual components, 2-phenylethanol and ethanol were detected at a similar level in volatile blends of all tested yeasts (Table 1). Ethyl acetate, ethyl propionate, and 3-methylbutanoic acid predominated in VOCs produced by *H. uvarum* and *M. pulcherrima*, but not *S. cerevisiae* and *S. paradoxus*. In addition, *H. uvarum* intensively produced 3-methylbutyl propionate, acetic acid, and 2-phenylethyl acetate, while *M. pulcherrima* produced propyl acetate. 3-Methyl-1-butanol and 2-methyl-1-propanol were more common in *S. cerevisiae* and *S. paradoxus* than *H. uvarum* and *M. pulcherrima* yeasts. Significant production of 3-methylbutyl acetate was detected in the VOCs emitted by all tested yeasts except *M. pulcherrima*.

**Figure 6:**
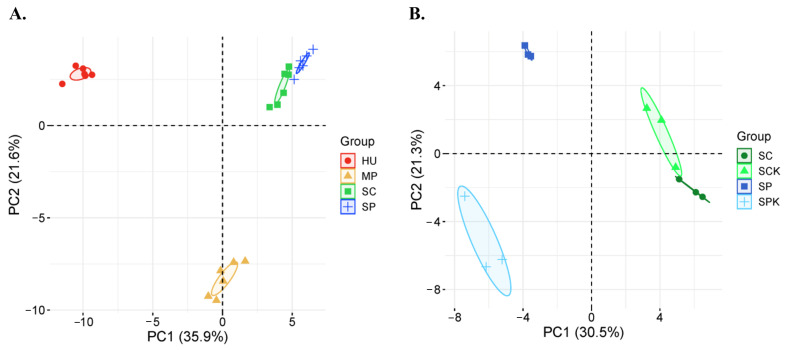
Yeast principal component analysis according to released volatile organic compounds. All tested yeast (**A**) and only *Saccharomyces* spp. strains (**B**). RC—rosehip, SA—rowanberry. K—killer yeast, K−—non-killer yeast. (A) HU—*H. uvarum*, MP—*M. pulcherrima*, SP—*S. paradoxus*, SC—*S. cerevisiae*; (B) SC, SP—non-killer yeasts, SCK, SPK—killer yeasts.

The principal component analysis (PCA) revealed distinct clustering of *H. uvarum*, *M. pulcherrima*, and both *Saccharomyces* strains based on their VOC profiles (*p*-value < 0.05), highlighting species-specific differences in VOC production (Figure 6A). To get a clearer view, *S. paradoxus* and *S. cerevisiae* are shown in a separate PCA plot (Figure 6B). The killer phenotype-possessing strains of *S. paradoxus* differed from the non-killer yeast of the same species, but representatives of *S. cerevisiae* yeasts remained close. Statistically significant differences were observed in the individual VOCs produced by killer and non-killer strains of *S. paradoxus*, but not *S. cerevisiae*. Killer yeasts of *S. paradoxus* produced significantly higher levels of ethyl acetate, ethanol, ethyl propionate, propyl acetate, 2-methyl-1-propanol, 3-methyl-1-butanol, and 3-methylbutanoic acid compared to non-killer strains (Mann–Whitney U test, *p*-value < 0.05) (Table S3).

## 4. Discussion

Rosehips and rowanberries are found in diverse and often challenging ecological environments, such as forests, mountain areas, and temperate regions across Europe and Asia [55,56]. These habitats expose the fruits to diverse climatic conditions and microbial interactions, fostering the development of bioactive compounds that influence the types of microorganisms capable of colonizing their surfaces [57,58]. Consequently, these fruits harbor a rich and diverse microbial population, including yeast species adapted to compete in nutrient-scarce and variable environments. These adaptations make yeasts from fruits excellent candidates for biocontrol applications. Multiple mechanisms, such as competition for nutrients and space, production of lytic enzymes and toxins, synthesis of VOCs and quorum sensing, etc., are involved in the execution of yeast-based biocontrol [2,41].

While the microbiota of rosehips and rowanberries has been explored in previous studies [8,9,10], the present work highlights yeast species inhabiting berries at low levels and focuses on their potential for biocontrol. Spontaneous fermentation was employed to enrich minor yeast species for analysis. It was identified that *H. uvarum* and *M. pulcherrima* were the most abundant yeast species, constituting approximately 70% of the total yeast population in spontaneous fermentations of rowanberries and rosehips. These data correspond to previous studies, where microbial profiling revealed that among cultivable yeasts distributed on the surface of these berries, the indicated species were also highly abundant [8]. Other yeast genera such as *Pichia*, *Saccharomyces*, and *Torulaspora* were not initially undetectable on raw rowanberries and rosehips [8] but became prominent after fermentation-based enrichment.

The identification of killer phenotypes across multiple genera reveals a widespread capacity for yeast-mediated microbial antagonism. The biocontrol abilities of many yeast species have been proven to correlate with the killer phenotype, and it is recognized as a highly important mechanism responsible for the antagonistic efficacy [59]. Among the 163 yeast isolates, 31 strains (belonging to almost all identified yeast species, such as *M. pulcherrima*, *H. uvarum*, *S. paradoxus*, *S. cerevisiae*, *P. anomala*, *P. kluyveri*, and *T. delbrueckii*) possessed killing activity. The killer phenomenon is widespread among numerous yeast genera, such as *Candida*, *Cryptococcus*, *Hanseniaspora*, *Kluyveromyces*, *Metschnikowia*, *Pichia*, *Saccharomyces*, *Torulaspora*, *Williopsis*, *Zygosaccharomyces*, etc., inhabiting different natural environments [11,60]. The killer determinants could be identified on the cytoplasmically inherited viral dsRNAs, linear plasmids, and chromosomal DNAs [61,62]. Various types of killer systems could be differentiated based on the genetic determination of killer toxins, their structure, and mechanisms of action. Killer strains are immune to their own toxin but remain susceptible to the action of other types of killer proteins [63,64]. However, the killing phenotype and spectrum of the antagonistic activity varies not only among different yeast species and types of killer yeast but also among the same killer type-possessing yeast strains [13]. Our study determined dsRNA-dependent *S. cerevisiae*, *S. paradoxus*, and *T. delbrueckii* killer phenotypes. Variations in the killing spectra and size of killer toxin-encoding M dsRNA pointed to the multiple types of killer toxins being produced by yeasts [13,14,16,18,65]. In our study, among *S. cerevisiae* yeasts, most likely K2 and Klus-type killer strains were isolated, and one *S. paradoxus* killer phenotype-possessing strain was attributed to the K66 type. The *T. delbrueckii* killer strain has a similarity to the Kbarr-1 type based on the size of M dsRNA; however, the antagonistic profile differs from that demonstrated by Kbarr-1 ability to kill all known *S. cerevisiae* killer strains [14]. Further studies, including viral dsRNA sequencing, are needed to characterize and confirm the specific killer types of all tested isolates. Our study determined that *S. cerevisiae* strains demonstrated the strongest yet low-spectrum antagonistic activity, particularly against other *Saccharomyces* spp. and *P. fermentans* yeasts, while *S. paradoxus* and *T. delbrueckii* killer strains showed low inhibitory capacity against non-killer and some killer type-possessing *S. cerevisiae* yeasts. Regardless of the limited antifungal activity of *S. cerevisiae* and *S. paradoxus* killer yeasts against closely related species and the narrow specificity spectrum to other yeasts [16,18,66], both species have high attractiveness for food and beverage preservation [11]. *T. delbrueckii* is often detected in spontaneous fermentations of fruits, beer, and wine, and belongs to the most used non-Saccharomyces yeast in winemaking [67]. Our study highlights the broad-spectrum antagonistic activity of *M. pulcherrima* and *P. anomala* isolates, noting that their killer phenotype does not appear to be associated with dsRNAs. The biocontrol ability of *M. pulcherrima* may be attributed to the DNA-encoded killer toxin [68] or the activity of pulcherrimin, a well-known compound for antimicrobial properties [43,69,70]. The high attractiveness of *Metschnikowia* spp. was demonstrated in winemaking to prevent spoilage by non-Saccharomyces yeasts. The profound antifungal and antibacterial activity increased their potential for the postharvest biocontrol of fruits and application in the food industry [43,71]. The killer phenotype in *P. anomala*, similar to *P. kluyveri*, is more likely to result from the chromosome-encoded killer toxin [21,72]. Killer yeasts, with their antimicrobial activity against susceptible microorganisms, have high potential in biological control by targeting postharvest fungal crop diseases, preserving fruit quality, and enhancing fermentation processes [25,26,73,74].

Killer toxin-producing yeasts efficiently control spoilage yeasts in the beverage and food industry [4,11,75]. *S. cerevisiae* K2 and Klus toxins and their secreting strains were validated as active players in winemaking, preventing the growth of contaminant yeasts [59]. In this study, the K2-type killer strain from rosehip berries was demonstrated to be active for the inhibition of potential fungal contaminants in a real food system—grape juice. Among numerous yeasts, species of *Candida*, *Pichia*, and *Saccharomyces* are reported as frequently present in fruit juice contamination [76]. K2-type killer toxins, due to high stability and activity at low pH [77], could be effectively used in acidic environments, especially in food fermentation [59]. In this study, the K2 toxin demonstrated profound activity against *S. cerevisiae* yeasts, while the viability of other fungal microorganisms from *Candida* and *Pichia* genera, potentially spoiling food products and posing a risk to human health, was reduced to a lesser extent. Therefore, the provided data prove the relevance of isolated killer yeasts for improving food safety.

VOC emission is one of the major antifungal mechanisms of antagonistic yeasts [78]. While previous research described volatile organic compounds synthesized by various yeasts [5,29,32,36], this study examines the profiles of VOCs in the context of the biocontrol ability of particular yeast species and their hosting plants. For VOC profiling, we chose *H. uvarum* and *M. pulcherrima* strains, dominant in spontaneous fermentations of rowanberries and rosehips and possessing biocontrol potential. Yeasts representing these species demonstrate antagonistic properties against fruit spoilage molds and fungi and are important for biocontrol [4,29,69,78,79,80]. The volatilomes of *S. paradoxus* and *S. cerevisiae* yeasts were analyzed to compare the VOC composition of dsRNA-encoded killer yeasts versus non-killer strains of the same species. Our study demonstrates that volatile profiles are yeast species-dependent and barely adhere to specific berries. Differences in VOCs of dsRNA-encoded killer strains compared to non-killer yeasts were established based on the analysis of *S. paradoxus* yeasts. In agreement with previous studies, conducted on sea buckthorn and sweet and sour cherry-inhabiting yeasts [29,34,35,36], esters and alcohols were the most abundant volatile metabolites collected from the headspace of *M. pulcherrima*, *H. uvarum*, and *Saccharomyces* spp. yeasts. The compounds of these groups have been associated with various biological activities, including antimicrobial properties relevant to biocontrol [4,29,78]. The most common esters detected in this study were ethyl acetate, 3-methylbutyl acetate, ethyl propionate, 3-methylbutyl propionate, and 2-phenylethyl acetate. Among alcohols, the most abundant were 2-methyl-1-propanol, 3-methyl-1-butanol, ethanol, and 2-phenylethanol. The amount of VOCs differed among the tested yeast species. Similar to our study, the prevalence of ethyl acetate in volatile blends of *H. uvarum* and *M. pulcherrima* yeasts isolated from sea buckthorn berries and sweet and sour cherries has been reported [35,36]. Ethyl propionate is mainly produced by *P. kudriavzevii* and *S. cerevisiae* yeasts found in fermented beverages [34,81]. Both esters effectively protect tomatoes from rotting agents or control gray mold in fruits [82,83]. 2-Phenylethyl acetate contributes to the aroma of fruity wines and has antifungal properties against *Aspergillus* spp. by inhibiting growth and toxin release [5,30,81,84]. The biocontrol ability of *W. anomalus* was attributed to the production of ethyl acetate and 2-phenyletanol [59]. Different yeasts, including *M. pulcherrima*, produce aromatic alcohols, which participate in biocontrol through quorum sensing [68]. It was demonstrated that the bioprotective effect of *H. uvarum* on lemon was induced by aromatic alcohol 2-phenyletanol synthesis [68]. In previous studies, 3-methylbutyl propionate was found to be emitted in large amounts by *P. kluyveri*, *T. delbrueckii*, *P. anomala*, *Cyberlinnera jadiniii*, *Lachancea thermotolerans*, and *H. uvarum* [30,34,35,84]. In contrast, 3-methylbutyl acetate is produced by *P. kudriavzevii*, *P. anomala*, *H. uvarum*, and *M. pulcherrima* [5,34,85,86]. 3-Methylbutyl propionate and 3-methylbutyl acetate were also detected in odor blends of sweet and sour cherry-inhabiting yeasts, and their emission was proposed as a predominant mechanism of antagonistic yeasts [35]. It is expected that 3-methylbutyl esters can induce intracellular ROS accumulation, which is one of the processes implicated in the antimicrobial antagonism of yeast strains [43,87]. Among alcohols, ethanol is widely used as a disinfectant, preservative, and food additive acting against a broad spectrum of microorganisms: bacteria, viruses, and yeasts [88,89,90,91,92]. 3-Methyl-1-butanol is naturally produced in significant quantities by *Kluyveromyces lactis*, *S. cerevisiae*, and *Pichia pastoris* yeasts and has demonstrated antifungal properties [93].

The killing phenotype-possessing *S. cerevisiae* and *S. paradoxus* yeasts were found in both berries tested. It should be noted that *S. cerevisiae* killer yeasts have stronger killing efficacy compared to *S. paradoxus*. However, when we compared the VOC profiles of killer versus non-killer yeasts, we found differences only in the case of *S. paradoxus*. This is likely due to the higher production of ethyl acetate, ethanol, ethyl propionate, propyl acetate, 2-methyl-1-propanol, 3-methyl-1-butanol, and 3-methylbutanoic acid. It appears feasible that *S. cerevisiae* killer yeasts actively produce toxins, which results in fewer volatile compounds being released. In contrast, the weaker antagonistic activity of *S. paradoxus* toxins may lead to compensation through the production of more bioactive volatile compounds. The relationship between emitted volatiles and mycotoxin production was revealed in the fungi *Aspergillus flavus*, and the possible links between toxin production and the emission of VOCs were hypothesized due to common parts of biosynthetic pathways [94,95]. Moreover, significant changes in VOC production and increased amounts of some aromatic compounds were observed in mixed inoculations of must with killer toxin-producing *T. delbrueckii* and *S. cerevisiae* strains, as compared to single inoculations [67]. However, more studies are needed to obtain an in-depth understanding of the links between yeast killer toxins and bioactive volatiles, determining antagonistic activity.

## 5. Conclusions

Overall, this study constituted the first comprehensive profiling of killer systems and volatile organic compounds of rowanberry and rosehip-inhabiting yeasts, offering novel insights into their biocontrol potential. The most common yeast species isolated from spontaneous fermentations of rowanberries and rosehips were *H. uvarum* and *M. pulcherrima*, making up roughly 70% of the total yeast population. The remaining 30% consisted of *Pichia*, *Saccharomyces*, *Torulaspora*, and *Candida* species. Approximately 20% of the isolated yeast strains demonstrated killing activity. The killing phenotype, encoded by dsRNA, was identified in *S. cerevisiae*, *S. paradoxus*, and *T. delbrueckii* yeasts and demonstrated that *S. cerevisiae* strains exhibit the strongest killing properties. *M. pulcherrima* and *P. anomala* displayed broad-spectrum biocontrol activity not linked to dsRNA, further emphasizing the diversity of antifungal mechanisms present in fruit-associated yeasts. The VOC profiles of yeast species were analyzed for the first time in the context of rowanberries and rosehips, revealing that VOC emissions are species-specific rather than defined by the host plant. Esters and alcohols were the most abundant groups of VOCs, many of which have established antimicrobial properties relevant to biocontrol. Comparative analysis revealed significant differences in VOC profiles between killer and non-killer *S. paradoxus* strains, highlighting the potential interplay between killer toxins and bioactive volatile compounds. This study also demonstrates the ability of *S. cerevisiae* K2-type killer strains to reduce fungal contaminants in grape juice, further validating their relevance for food safety applications.

## Figures and Tables

**Table 1:** The most abundant compounds emitted by *H. uvarum*, *M. pulcherrima*, *S. paradoxus*, and *S. cerevisiae* yeasts isolated from spontaneous fermentation of rowanberries and rosehips.

No	Compound	CAS No	RI	GR	Control	HU SA	HU RC	MP SA	MP RC	SP SA	SP RC	SC SA	SC RC
1	Ethyl acetate	141-78-6	nd	ES	15.1 ± 12.3	131.5 ± 51.7	110.3 ± 14.2	280.5 ± 12.7	220.3 ± 55.8	20.9 ± 15.9 ns	30.4 ± 17.2 ns	51.2 ± 24.9 ns	33.6 ± 23.6 ns
2	Ethanol	64-17-5	nd	OH	6.7 ± 3.7	27.6 ± 3.7	24.9 ± 2.5	34.9 ± 0.7	42.0 ± 2.9	31.2 ± 6.5	36.2 ± 5.8	33.9 ± 2.8	38.4 ± 1.5
3	Ethyl propionate	1105-37-3	nd	ES	4.6 ± 1.7	25.3 ± 7.9	25.0 ± 3.6	32.8 ± 5.4	24.9 ± 9.9	8.9 ± 1.2	11.2 ± 2.5	8.4 ± 2.9 ns	11.8 ± 5.1
4	Propyl acetate	109-60-4	nd	ES	0.7 ± 0.6	6.1 ± 3.7	4.3 ± 0.9	15.3 ± 1.5	11.6 ± 3.5	1.5 ± 0.9 ns	2.2 ± 0.3	4.2 ± 1.3	2.7 ± 1.6 ns
5	2-Methyl-1-propanol	78-83-1	1094	OH	0	3.1 ± 1.0	6.3 ± 3.2	5.8 ± 0.6	14.5 ± 3.7	12.3 ± 3.8	13.6 ± 3.8	10.2 ± 1.7	10.8 ± 1.5
6	3-Methylbutyl acetate	123-92-2	1112	ES	1.5 ± 1.3	86.0 ± 20.2	88.2 ± 15.4	23.5 ± 2.3	25.3 ± 6.2	63.4 ± 2.8	67.6 ± 10.1	56.1 ± 8.4	59.5 ± 9.2
7	3-Methylbutyl propionate	105-68-0	1180	ES	0	26.3 ± 8.6	23.5 ± 5.2	2.0 ± 0.3	1.1 ± 0.6	5.8 ± 1.3	12.8 ± 7.9	8.6 ± 2.6	8.7 ± 2.8
8	3-Methyl-1-butanol	123-51-3	1211	OH	1.2 ± 1.2	59.3 ± 7.8	62.8 ± 3.4	53.9 ± 3.6	78.5 ± 11.2	118.7 ± 6.6	130.1 ± 8.1	157.8 ± 7.0	161.7 ± 19.5
9	Acetic acid	64-19-7	1441	FA	0.6 ± 1.1	23.9 ± 6.1	23.8 ± 6.0	2.5 ± 1.8	0.3 ± 0.5 ns	0.9 ± 0.1 ns	0.7 ± 0.2 ns	0.7 ± 0.1 ns	0.6 ± 0.1 ns
10	3-Methylbutanoic acid	503-74-2	1662	FA	2.8 ± 2.3	39.2 ± 3.5	41.7 ± 3.0	22.7 ± 3.6	11.1 ± 9.3 ns	0.9 ± 0.3	0.9 ± 0.4 ns	4.8 ± 2.1 ns	4.6 ± 1.3 ns
11	2-Phenylethyl acetate	103-45-7	1804	AR, ES	0	22.1 ± 8.1	19.9 ± 3.6	2.9 ± 1.1	4.6 ± 3.8	2.7 ± 1.4	1.6 ± 0.4	5.8 ± 1.1	5.3 ± 1.7
12	2-Phenylethanol	60-12-8	1895	AR, OH	1.9 ± 1.1	44.1 ± 4.9	45.7 ± 2.6	29.5 ± 7.8	34.0 ± 6.3	53.4 ± 10.9	43.9 ± 4.4	63.4 ± 4.2	66.2 ± 12.5

HU—*H. uvarum*, MP—*M. pulcherrima*, SP—*S. paradoxus*, SC—*S. cerevisiae*; SA—rowanberry (*Sorbus aucuparia* L.), RC—rosehip (*Rosa canina* L.). Data showed as mean ± standard deviation of the mean (means are the amounts expressed as areas under the chromatographic peaks and have to be read as number times 1,000,000). CAS No—chemical abstract service number; RI—retention index; GR—group of chemical compounds. nd—not determined. ES—ester, OH—alcohol, AR—aromatic, FA—fatty acid. ns—not significantly different compared to control (Mann–Whitney U test, *p* < 0.05); three different isolates of each yeast species have been used.

## Data Availability

Data is contained within the article and Supplementary Materials, further inquiries can be directed to the corresponding author.

## Supplementary Materials

The following supporting information is available online at https://www.mdpi.com/article/10.3390/foods14020288/s1, Table S1: Zone inhibition assay data of yeast strains isolated from spontaneous fermentations of rosehips and rowanberries, Table S2: Volatile organic compounds produced by *H. uvarum*, *M. pulcherrima*, *S. paradoxus*, and *S. cerevisiae*, isolated from spontaneous fermentation of rowanberries and rosehips using the SPME technique, Table S3: Volatile organic compound raw data (area plots) for *H. uvarum*, *M. pulcherrima*, *S. paradoxus*, and *S. cerevisiae*.

## References

[1] Agirman B., Carsanba E., Settanni L., Erten H. (2023). Exploring Yeast-Based Microbial Interactions: The next Frontier in Postharvest Biocontrol. Yeast.

[2] Kowalska J., Krzymińska J., Tyburski J. (2022). Yeasts as a Potential Biological Agent in Plant Disease Protection and Yield Improvement—A Short Review. Agriculture.

[3] Ma Y., Wu M., Qin X., Dong Q., Li Z. (2023). Antimicrobial Function of Yeast against Pathogenic and Spoilage Microorganisms via Either Antagonism or Encapsulation: A Review. Food Microbiol..

[4] Freimoser F.M., Rueda-Mejia M.P., Tilocca B., Migheli Q. (2019). Biocontrol Yeasts: Mechanisms and Applications. World J. Microbiol. Biotechnol..

[5] Oufensou S., Ul Hassan Z., Balmas V., Jaoua S., Migheli Q. (2023). Perfume Guns: Potential of Yeast Volatile Organic Compounds in the Biological Control of Mycotoxin-Producing Fungi. Toxins.

[6] Parafati L., Vitale A., Restuccia C., Cirvilleri G. (2015). Biocontrol Ability and Action Mechanism of Food-Isolated Yeast Strains against *Botrytis cinerea* Causing Post-Harvest Bunch Rot of Table Grape. Food Microbiol..

[7] Sui Y., Wisniewski M., Droby S., Liu J. (2015). Responses of Yeast Biocontrol Agents to Environmental Stress. Appl. Environ. Microbiol..

[8] Vepštaitė-Monstavičė I., Lukša J., Strazdaitė-Žielienė Ž., Serva S., Servienė E. (2024). Distinct Microbial Communities Associated with Health-relevant Wild Berries. Environ. Microbiol. Rep..

[9] Maksimova I.A., Yurkov A.M., Chernov I.Y. (2009). Spatial Structure of Epiphytic Yeast Communities on Fruits of *Sorbus aucuparia* L. Biol. Bull..

[10] Rovná K., Ivanišová E., Žiarovská J., Ferus P., Terentjeva M., Kowalczewski P.Ł., Kačániová M. (2020). Characterization of *Rosa canina* Fruits Collected in Urban Areas of Slovakia. Genome Size, IPBS Profiles and Antioxidant and Antimicrobial Activities. Molecules.

[11] Billerbeck S., Walker R.S.K., Pretorius I.S. (2024). Killer Yeasts: Expanding Frontiers in the Age of Synthetic Biology. Trends Biotechnol..

[12] Büyüksırıt Bedir T., Kuleaşan H. (2021). A Natural Approach, the Use of Killer Toxin Produced by *Metschnikowia pulcherrima* in Fresh Ground Beef Patties for Shelf Life Extention. Int. J. Food Microbiol..

[13] Chang S.L., Leu J.Y., Chang T.H. (2015). A Population Study of Killer Viruses Reveals Different Evolutionary Histories of Two Closely Related *Saccharomyces Sensu Stricto* Yeasts. Mol. Biol..

[14] Ramírez M., Velázquez R., López-Piñeiro A., Naranjo B., Roig F., Llorens C. (2017). New Insights into the Genome Organization of Yeast Killer Viruses Based on “Atypical” Killer Strains Characterized by High-Throughput Sequencing. Toxins.

[15] Rodríguez-Cousiño N., Gómez P., Esteban R. (2017). Variation and Distribution of L-A Helper Totiviruses in *Saccharomyces Sensu Stricto* Yeasts Producing Different Killer Toxins. Toxins.

[16] Vepštaitė-Monstavičė I., Lukša J., Konovalovas A., Ežerskytė D., Stanevičienė R., Strazdaitė-Žielienė Ž., Serva S., Servienė E. (2018). *Saccharomyces paradoxus* K66 Killer System Evidences Expanded Assortment of Helper and Satellite Viruses. Viruses.

[17] Villalba M.L., Susana Sáez J., del Monaco S., Lopes C.A., Sangorrín M.P. (2016). TdKT, a New Killer Toxin Produced by *Torulaspora delbrueckii* Effective against Wine Spoilage Yeasts. Int. J. Food Microbiol..

[18] Rodriguez-Cousino N., Gomez P., Esteban R. (2022). Expression of the K74 Killer Toxin from *Saccharomyces paradoxus* Is Modulated by the Toxin-Encoding M74 Double- Stranded RNA 59 Untranslated Terminal Region. Appl. Environ. Microbiol..

[19] Lukša J., Podoliankaitė M., Vepštaitė I., Strazdaitė-Žielienė Ž., Urbonavičius J., Servienė E. (2015). Yeast β-1,6-Glucan Is a Primary Target for the *Saccharomyces cerevisiae* K2 Toxin. Eukaryot. Cell.

[20] Martinac B., Zhu H., Kubalski A., Zhou X.L., Culbertson M., Bussey H., Kung C. (1990). Yeast K1 Killer Toxin Forms Ion Channels in Sensitive Yeast Spheroplasts and in Artificial Liposomes. Proc. Natl. Acad. Sci. USA.

[21] Schmitt M.J., Breinig F. (2002). The Viral Killer System in Yeast: From Molecular Biology to Application. FEMS Microbiol. Rev..

[22] Prins R.C., Billerbeck S. (2024). The Signal Sequence of Yeast Killer Toxin K2 Confers Producer Self-Protection and Allows Conversion into a Modular Toxin-Antitoxin System. Cell Rep..

[23] Becker B., Schmitt M.J. (2017). Yeast Killer Toxin K28: Biology and Unique Strategy of Host Cell Intoxication and Killing. Toxins.

[24] Reiter J., Herker E., Madeo F., Schmitt M.J. (2005). Viral Killer Toxins Induce Caspase-Mediated Apoptosis in Yeast. J. Cell Biol..

[25] Díaz M.A., Pereyra M.M., Picón-Montenegro E., Meinhardt F., Dib J.R. (2020). Killer Yeasts for the Biological Control of Postharvest Fungal Crop Diseases. Microorganisms.

[26] Di Canito A., Mateo-Vargas M.A., Mazzieri M., Cantoral J., Foschino R., Cordero-Bueso G., Vigentini I. (2021). The Role of Yeasts as Biocontrol Agents for Pathogenic Fungi on Postharvest Grapes: A Review. Foods.

[27] Klassen R., Schaffrath R., Buzzini P., Ganter P., Buzzini P., Lachance M.A., Yurkov A. (2017). Antagonistic Interactions and Killer Yeasts. Yeasts in Natural Ecosystems: Ecology.

[28] Schaffrath R., Meinhardt F., Klassen R., Anke T., Schüffler A. (2018). Yeast Killer Toxins: Fundamentals and Applications. Physiology and Genetics: Selected Basic and Applied Aspects.

[29] Contarino R., Brighina S., Fallico B., Cirvilleri G., Parafati L., Restuccia C. (2019). Volatile Organic Compounds (VOCs) Produced by Biocontrol Yeasts. Food Microbiol..

[30] Farbo M.G., Urgeghe P.P., Fiori S., Marcello A., Oggiano S., Balmas V., Hassan Z.U., Jaoua S., Migheli Q. (2018). Effect of Yeast Volatile Organic Compounds on Ochratoxin A-Producing *Aspergillus carbonarius* and *A. Ochraceus*. Int. J. Food Microbiol..

[31] Sater H.M., Bizzio L.N., Tieman D.M., Muñoz P.D. (2020). A Review of the Fruit Volatiles Found in Blueberry and Other *Vaccinium* Species. J. Agric. Food Chem..

[32] Karsli A., Şahin Y.S. (2021). The Role of Fungal Volatile Organic Compounds (FVOCs) in Biological Control. Türkiye Biyolojik Mücadele Derg..

[33] Parafati L., Vitale A., Restuccia C., Cirvilleri G. (2017). Performance Evaluation of Volatile Organic Compounds by Antagonistic Yeasts Immobilized on Hydrogel Spheres against Gray, Green and Blue Postharvest Decays. Food Microbiol..

[34] Mozūraitis R., Aleknavičius D., Vepštaitė-Monstavičė I., Stanevičienė R., Emami S.N., Apšegaitė V., Radžiutė S., Blažytė-Čereškienė L., Servienė E., Būda V. (2020). *Hippophae rhamnoides* Berry Related *Pichia kudriavzevii* Yeast Volatiles Modify Behaviour of *Rhagoletis batava* Flies. J. Adv. Res..

[35] Mozūraitis R., Apšegaitė V., Radžiutė S., Aleknavičius D., Būdienė J., Stanevičienė R., Blažytė-Čereškienė L., Servienė E., Būda V. (2022). Volatiles Produced by Yeasts Related to *Prunus avium* and *P. cerasus* Fruits and Their Potentials to Modulate the Behaviour of the Pest *Rhagoletis cerasi* Fruit Flies. J. Fungi.

[36] Lukša J., Vepštaitė-Monstavičė I., Apšegaitė V., Blažytė-Čereškienė L., Stanevičienė R., Strazdaitė-Žielienė Ž., Ravoitytė B., Aleknavičius D., Būda V., Mozūraitis R. (2020). Fungal Microbiota of Sea Buckthorn Berries at Two Ripening Stages and Volatile Profiling of Potential Biocontrol Yeasts. Microorganisms.

[37] Huang R., Li G.Q., Zhang J., Yang L., Che H.J., Jiang D.H., Huang H.C. (2011). Control of Postharvest Botrytis Fruit Rot of Strawberry by Volatile Organic Compounds of *Candida intermedia*. Phytopathology.

[38] Lemos Junior W.J.F., Binati R.L., Felis G.E., Slaghenaufi D., Ugliano M., Torriani S. (2020). Volatile Organic Compounds from *Starmerella bacillaris* to Control Gray Mold on Apples and Modulate Cider Aroma Profile. Food Microbiol..

[39] Huang R., Che H.J., Zhang J., Yang L., Jiang D.H., Li G.Q. (2012). Evaluation of *Sporidiobolus pararoseus* Strain YCXT3 as Biocontrol Agent of *Botrytis cinerea* on Post-Harvest Strawberry Fruits. Biol. Control.

[40] Di Francesco A., Ugolini L., Lazzeri L., Mari M. (2015). Production of Volatile Organic Compounds by *Aureobasidium pullulans* as a Potential Mechanism of Action against Postharvest Fruit Pathogens. Biol. Control.

[41] Aragno J., Fernandez-Valle P., Thiriet A., Grondin C., Legras J.L., Camarasa C., Bloem A. (2024). Two-Stage Screening of *Metschnikowia* spp. Bioprotective Properties: From Grape Juice to Fermented Must by *Saccharomyces cerevisiae*. Microorganisms.

[42] Moore G.G., Lebar M.D., Carter-Wientjes C.H. (2022). Cumulative Effects of Non-Aflatoxigenic *Aspergillus flavus* Volatile Organic Compounds to Abate Toxin Production by Mycotoxigenic Aspergilli. Toxins.

[43] Sipiczki M. (2020). *Metschnikowia pulcherrima* and Related Pulcherrimin-Producing Yeasts: Fuzzy Species Boundaries and Complex Antimicrobial Antagonism. Microorganisms.

[44] Lukša J., Ravoitytė B., Konovalovas A., Aitmanaitė L., Butenko A., Yurchenko V., Serva S., Servienė E. (2017). Different Metabolic Pathways Are Involved in Response of *Saccharomyces cerevisiae* to L-A and M Viruses. Toxins.

[45] Gulbiniene G., Kondratiene L., Jokantaite T., Serviene E., Melvydas V., Petkuniene G. (2004). Killer Yeast Strains in Wine Yeast Population. Food Technol. Biotechnol..

[46] Vepštaitė-Monstavičė I., Lukša J., Stanevičienė R., Strazdaitė-Žielienė Ž., Yurchenko V., Serva S., Servienė E. (2018). Distribution of Apple and Blackcurrant Microbiota in Lithuania and the Czech Republic. Microbiol. Res..

[47] Stanevičienė R., Lukša J., Strazdaitė-Žielienė Ž., Ravoitytė B., Losinska-Sičiūnienė R., Mozūraitis R., Servienė E. (2021). Mycobiota in the Carposphere of Sour and Sweet Cherries and Antagonistic Features of Potential Biocontrol Yeasts. Microorganisms.

[48] Servienė E., Lukša J., Orentaitė I., Lafontaine D.L.J., Urbonavičius J. (2012). Screening the Budding Yeast Genome Reveals Unique Factors Affecting K2 Toxin Susceptibility. PLoS ONE.

[49] Fried H.M., Fink G.R. (1978). Electron Microscopic Heteroduplex Analysis of “Killer” Double-Stranded RNA Species from Yeast. Proc. Natl. Acad. Sci. USA.

[50] Grybchuk D., Akopyants N.S., Kostygov A.Y., Konovalovas A., Lye L.-F., Dobson D.E., Zangger H., Fasel N., Butenko A., Frolov A.O. (2018). Viral Discovery and Diversity in Trypanosomatid Protozoa with a Focus on Relatives of the Human Parasite *Leishmania*. Proc. Natl. Acad. Sci. USA.

[51] Van Rossum G., Drake F.L. (2015). The Python Language Reference.

[52] Wilcox R.R. (2011). Introduction to Robust Estimation and Hypothesis Testing.

[53] Tang D., Chen M., Huang X., Zhang G., Zeng L., Zhang G., Wu S., Wang Y. (2023). SRplot: A Free Online Platform for Data Visualization and Graphing. PLoS ONE.

[54] Ramírez M., Velázquez R., Maqueda M., López-Piñeiro A., Ribas J.C. (2015). A New Wine *Torulaspora delbrueckii* Killer Strain with Broad Antifungal Activity and Its Toxin-Encoding Double-Stranded RNA Virus. Front. Microbiol..

[55] Aurori M., Niculae M., Hanganu D., Pall E., Cenariu M., Vodnar D.C., Fiţ N., Andrei S. (2024). The Antioxidant, Antibacterial and Cell-Protective Properties of Bioactive Compounds Extracted from Rowanberry (*Sorbus aucuparia* L.) Fruits In Vitro. Plants.

[56] Igual M., García-Herrera P., Cámara R.M., Martínez-Monzó J., García-Segovia P., Cámara M. (2022). Bioactive Compounds in Rosehip (*Rosa canina*) Powder with Encapsulating Agents. Molecules.

[57] Arvinte O.M., Senila L., Becze A., Amariei S. (2023). Rowanberry—A Source of Bioactive Compounds and Their Biopharmaceutical Properties. Plants.

[58] Winther K., Campbell-Tofte J., Vinther Hansen A.S. (2016). Bioactive Ingredients of Rose Hips (*Rosa canina* L.) with Special Reference to Antioxidative and Anti-Inflammatory Properties: In Vitro Studies. Botanics.

[59] Muccilli S., Restuccia C. (2015). Bioprotective Role of Yeasts. Microorganims.

[60] Bajaj B.K., Singh S., Satyanarayana T., Kunze G. (2017). Biology of Killer Yeast and Technological Implications. Yeast Diversity in Human Welfare.

[61] Vijayraghavan S., Kozmin S.G., Strope P.K., Skelly D.A., Magwene P.M., Dietrich F.S., McCusker J.H. (2023). RNA Viruses, M Satellites, Chromosomal Killer Genes, and Killer/Nonkiller Phenotypes in the 100-Genomes *S. cerevisiae* Strains. G3 Genes. Genom. Genet..

[62] Travers-Cook T.J., Jokela J., Buser C.C. (2023). The Evolutionary Ecology of Fungal Killer Phenotypes. Proc. Roy. Soc. B-Biol. Sci..

[63] Boynton P.J. (2019). The Ecology of Killer Yeasts: Interference Competition in Natural Habitats. Yeast.

[64] Giovati L., Ciociola T., De Simone T., Conti S., Magliani W. (2021). *Wickerhamomyces* Yeast Killer Toxins’ Medical Applications. Toxins.

[65] Rodríguez-Cousiño N., Maqueda M., Ambrona J., Zamora E., Esteban R., Ramírez M. (2011). A New Wine *Saccharomyces cerevisiae* Killer Toxin (Klus), Encoded by a Double-Stranded RNA Virus, with Broad Antifungal Activity Is Evolutionarily Related to a Chromosomal Host Gene. Appl. Environ. Microbiol..

[66] Crabtree A.M., Taggart N.T., Lee M.D., Boyer J.M., Rowley P.A. (2023). The Prevalence of Killer Yeasts and Double-Stranded RNAs in the Budding Yeast *Saccharomyces cerevisiae*. FEMS Yeast Res..

[67] Velazquez R., Zamora E., Alvarez M.L., Ramirez M. (2019). Using *Torulaspora delbrueckii* Killer Yeasts in the Elaboration of Base Wine and Traditional Sparkling Wine. Int. J. Food Microbiol..

[68] Puyo M., Simonin S., Bach B., Klein G., Alexandre H., Tourdot-Maréchal R. (2023). Bio-protection in Enology by *Metschnikowia pulcherrima*: From Field Results to Scientific Inquiry. Front. Microbiol..

[69] Sipiczki M. (2006). *Metschnikowia* Strains Isolated from Botrytized Grapes Antagonize Fungal and Bacterial Growth by Iron Depletion. Appl. Environ. Microbiol..

[70] Gore-Lloyd D., Sumann I., Brachmann A.O., Schneeberger K., Ortiz-Merino R.A., Moreno-Beltrán M., Schläfli M., Kirner P., Santos Kron A., Rueda-Mejia M.P. (2019). Snf2 Controls Pulcherriminic Acid Biosynthesis and Antifungal Activity of the Biocontrol Yeast *Metschnikowia pulcherrima*. Mol. Microbiol..

[71] Hicks R.H., Moreno-Beltrán M., Gore-Lloyd D., Chuck C.J., Henk D.A. (2021). The Oleaginous Yeast *Metschnikowia pulcherrima* Dislays Killer Activity against Avian-Derived Pathogenic Bacteria. Biology.

[72] Schneider J., Rupp O., Trost E., Jaenicke S., Passoth V., Goesmann A., Tauch A., Brinkrolf K. (2012). Genome Sequence of *Wickerhamomyces Anomalus* DSM 6766 Reveals Genetic Basis of Biotechnologically Important Antimicrobial Activities. FEMS Yeast Res..

[73] Grzegorczyk M., Żarowska B., Restuccia C., Cirvilleri G. (2017). Postharvest Biocontrol Ability of Killer Yeasts against *Monilinia fructigena* and *Monilinia fructicola* on Stone Fruit. Food Microbiol..

[74] Villalba M.L., Mazzucco M.B., Lopes C.A., Ganga M.A., Sangorrín M.P. (2020). Purification and Characterization of *Saccharomyces eubayanus* Killer Toxin: Biocontrol Effectiveness against Wine Spoilage Yeasts. Int. J. Food Microbiol..

[75] Belda I., Ruiz J., Alonso A., Marquina D., Santos A. (2017). The Biology of *Pichia membranifaciens* Killer Toxins. Toxins.

[76] da Cruz Almeida E.T., de Medeiros Barbosa I., Tavares J.F., Barbosa-Filho J.M., Magnani M., de Souza E.L. (2018). Inactivation of Spoilage Yeasts by *Mentha spicata* L. and M. *× villosa* Huds. Essential Oils in Cashew, Guava, Mango, and Pineapple Juices. Front. Microbiol..

[77] Lukša J., Serva S., Servienė E. (2017). *Saccharomyces cerevisiae* K2 Toxin Requires Acidic Environment for Unidirectional Folding Into Active State. Mycoscience.

[78] Zhao X., Zhou J., Tian R., Liu Y. (2022). Microbial Volatile Organic Compounds: Antifungal Mechanisms, Applications, and Challenges. Front. Microbiol..

[79] Liu H.M., Guo J.H., Cheng Y.J., Luo L., Liu P., Wang B.Q., Deng B.X., Long C.A. (2010). Control of Gray Mold of Grape by *Hanseniaspora uvarum* and Its Effects on Postharvest Quality Parameters. Ann. Microbiol..

[80] Janisiewicz W.J., Tworkoski T.J., Kurtzman C.P. (2001). Biocontrol Potential of *Metchnikowia pulcherrima* Strains Against Blue Mold of Apple. Phytopatology.

[81] Rosend J., Kuldjärv R., Rosenvald S., Paalme T. (2019). The Effects of Apple Variety, Ripening Stage, and Yeast Strain on the Volatile Composition of Apple Cider. Heliyon.

[82] Liu Z., Tian J., Yan H., Li D., Wang X., Liang W., Wang G. (2022). Ethyl Acetate Produced by *Hanseniaspora uvarum* Is a Potential Biocontrol Agent against Tomato Fruit Rot Caused by *Phytophthora nicotianae*. Front. Microbiol..

[83] Ruiz-Moyano S., Hernández A., Galvan A.I., Córdoba M.G., Casquete R., Serradilla M.J., Martín A. (2020). Selection and Application of Antifungal VOCs-Producing Yeasts as Biocontrol Agents of Grey Mould in Fruits. Food Microbiol..

[84] Hua S.S.T., Beck J.J., Sarreal S.B.L., Gee W. (2014). The Major Volatile Compound 2-Phenylethanol from the Biocontrol Yeast, *Pichia Anomala*, Inhibits Growth and Expression of Aflatoxin Biosynthetic Genes of *Aspergillus flavus*. Mycotoxin Res..

[85] Di Francesco A., Zajc J., Gunde-Cimerman N., Aprea E., Gasperi F., Placì N., Caruso F., Baraldi E. (2020). Bioactivity of Volatile Organic Compounds by *Aureobasidium* Species against Gray Mold of Tomato and Table Grape. World J. Microbiol. Biotechnol..

[86] Khunnamwong P., Lertwattanasakul N., Jindamorakot S., Suwannarach N., Matsui K., Limtong S. (2020). Evaluation of Antagonistic Activity and Mechanisms of Endophytic Yeasts against Pathogenic Fungi Causing Economic Crop Diseases. Folia Microbiol..

[87] Sanchez-Hernandez E., Gonzalez-Garcia V., Palacio-Bielsa A., Casanova-Gascon J., Navas-Gracia L.M., Martin-Gil J., Martin-Ramos P. (2023). Phytochemical Constituents and Antimicrobial Activity of *Euphorbia serrata* L. Extracts for *Borago officinalis* L. Crop Protection. Horticulture.

[88] Le Dare B., Lagente V., Gicquel T. (2019). Ethanol and Its Metabolites: Update on Toxicity, Benefits, and Focus on Immunomodulatory Effects. Drug Metab. Rev..

[89] Kim Y.H., Kim J.H., Jin H.J., Lee S.Y. (2013). Antimicrobial Activity of Ethanol Extracts of *Laminaria japonica* against Oral Microorganisms. Anaerobe.

[90] Turu D., Bozyel M.E., Candan K., Yakan M.A., Eray Bozyel M., Benek A., Canli K. (2020). In Vitro Antimicrobial and Antioxidant Activities of *Pyracantha coccinea* Fruits Ethanol Extract. Int. J. Acad. Multidiscip. Res..

[91] Watts S., Ramstedt M., Salentinig S. (2021). Ethanol Inactivation of Enveloped Viruses: A Structural and Surface Chemistry Insight on Phi6. J. Phys. Chem. Lett..

[92] Peters B.M., Ward R.M., Rane H.S., Lee S.A., Noverr M.C. (2013). Efficacy of Ethanol against *Candida albicans* and *Staphylococcus aureus* Polymicrobial Biofilms. Antimicrob. Agents Chemother..

[93] Fialho M.B., de Moraes M.H.D., Tremocoldi A.R., Pascholati S.F. (2011). Potential of Antimicrobial Volatile Organic Compounds to Control *Sclerotinia sclerotiorum* in Bean Seeds. Pesqui. Agropecu. Bras..

[94] Josselin L., De Clerck C., De Boevre M., Moretti A., Jijakli M.H., Soyeurt H., Fauconnier M.-L. (2021). Volatile Organic Compounds Emitted by *Aspergillus flavus* Strains Producing or Not Aflatoxin B1. Toxins.

[95] Caceres I., Al Khoury A., El Khoury R., Lorber S., Oswald I.P., El Khoury A., Atoui A., Puel O., Bailly J.D. (2020). Aflatoxin Biosynthesis and Genetic Regulation: A Review. Toxins.

